# Norovirus Variant GII.4/Sydney/2012, Bangladesh

**DOI:** 10.3201/eid1908.130227

**Published:** 2013-08

**Authors:** Mustafizur Rahman, Shamsun Nahar, Mokibul Hassan Afrad, Abu S.G. Faruque, Tasnim Azim

**Affiliations:** International Centre for Diarrhoeal Disease Research, Bangladesh, Dhaka, Bangladesh (M. Rahman, M.H. Afrad, A.S.G. Faruque, T. Azim);; Jahangirnagar University, Dhaka (S. Nahar)

**Keywords:** norovirus, GII.4/Sydney/2012, Bangladesh, viruses, enteric infections, gastroenteritis, foodborne infections, waterborne infections, diarrhea

**To the Editor:** Noroviruses (NoVs) are the most common cause of foodborne and waterborne outbreaks of gastroenteritis in persons from all age groups in industrialized and developing countries (*1*). Although NoV outbreaks occur throughout the year, activity increases in the winter months, especially in the countries with a temperate climate. As expected, during the last few months of 2012, outbreaks of NoV gastroenteritis markedly increased in Europe and the United States (*2*–*4*). These increases corresponded with the emergence of a variant of genotype GII.4, Sydney/2012, which was first reported from Australia in March 2012 and, subsequently, in the United States, Belgium, Denmark, Scotland, and Japan (*2*,*5*–*7*).

We identified the NoV GII.4 variant Sydney/2012 through hospital surveillance on diarrhea etiology in Bangladesh in December 2011 and then throughout 2012. These strains came from 3 hospitals in Dhaka, Matlab, and Mirzapur, where ≈150,000 patients with diarrhea are treated annually. We randomly selected 795 fecal specimens from patients of all ages who sought treatment for diarrhea in these hospitals during 2010–2012 and detected NoV RNA in 90 (33.6%), 72 (27.9%), and 92 (34.2%) samples in 2010, 2011, and 2012, respectively, by performing real-time PCR (*8*). For characterization, we amplified and sequenced 108 samples on the basis of the capsid genes (*9*).

Ages of diarrhea patients with NoV infection ranged from 1 month to 91 years (median 15 months; mean 11.9 years). Most (66%) NoV-positive patients were <5 years of age. Infection rates were lowest in patients <3 months (2.1%) and 5–18 years (2.5%) of age. A high number of NoV infections were recorded in adults (28.8% in patients >18 years of age). NoVs were detected throughout the year, and no clear seasonal peaks were observed.

Overall, GII was the most predominant genogroup (66.1%), followed by GI (18.1%) and GIV (3.9%). Mixed infections were detected in 11.8% of samples. We observed a high diversity in the GII genogroup and identified at least 11 different genotypes within the group, in which GII.4 constituted 30.1% of all GII strains. Until December 2011, the GII.4 variant NewOrleans/2009 was the most predominant strain (Figure). However, the new GII.4 variant, Sydney/2012, replaced the old variant and appeared as the dominant strain in 2012. We constructed a phylogenetic tree on the basis of 1,026 bases around the junction region of *pol* and *cap* genes, and it revealed that the newly identified variant has evolved from previous NoV GII.4 variants Apeldoorn/2007 and NewOrleans/2009 (data not shown).

**Figure:**
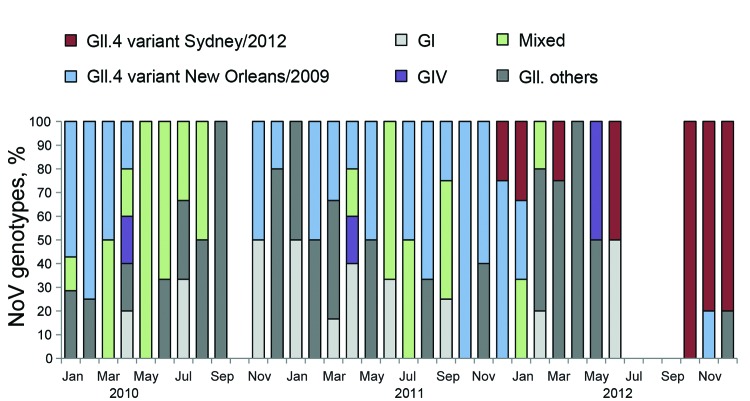
Distribution of 108 norovirus (NoV) genotypes in Bangladesh, 2010–2012. Bar chart shows the percentage of NoV genotypes. Mixed genotypes comprise NoV GI and GII. GI comprises GI.1, GI.3, GI.4, GI.5, and GI.9. GII.others comprises GII.2, GII.3, GII.4, GII.6, GII.10, GII.13, GII.16, GII.17, and GII.21.

NoVs, old and new, remain a substantial threat to human health, with a new variant emerging every 2–3 years. The Sydney/2012 strain appears to have replaced the previously predominant strain, but its clinical effects and epidemiology are largely unknown and warrant further investigation.

## References

[1] Green KY. Caliciviridae: the noroviruses. In: Fields BN, Knipe DM, Howley PM, Griffin DE, Lamb RA, Martin MA, et al, editors. Fields virology, 5th ed. Philadelphia: Lippincott, Williams & Wilkins; 2007. p. 949–79.

[2] van Beek J, Ambert-Balay K, Botteldoorn N, Eden J, Fonager J, Hewitt J, Indications for worldwide increased norovirus activity associated with emergence of a new variant of genotype II.4, late 2012. Euro Surveill. 2013;18:8–9 . 23305715

[3] Bennett S, Maclean A, Miller R, Aitken C, Gunson R. Increased norovirus activity in Scotland in 2012 is associated with the emergence of a new norovirus GII.4 variant. Euro Surveill. 2013;18:20349 . 23324428

[4] Fonager J, Hindbæk LS, Fischer TK. Rapid emergence and antigenic diversification of the norovirus 2012 Sydney variant in Denmark, October to December, 2012. Euro Surveill. 2013;18:20413 . 23470017

[5] National Institute of Infectious Diseases. Japan. Flash report of norovirus in Japan. Tokyo [cited 10 Jan 2013]. http://www.nih.go.jp/niid/en/iasr-noro-e.html

[6] White PA, Eden JS, Hansman GS. Molecular epidemiology of noroviruses and sapoviruses and their role in Australian outbreaks of acute gastroenteritis. Microbiology Australia. 2012;33:70–3 [cited 2013 Jun 5]. http://journalscambridgemedia.com.au/UserDir/ CambridgeJournal/Articles/08white409.pdf

[7] Centers for Disease Control and Prevention. Emergence of new norovirus strain GII.4 Sydney—United States, 2012. MMWR Morb Mortal Wkly Rep. 2013;62:55 . 23344699 PMC4604874

[8] Trujillo AA, McCaustland KA, Zheng DP, Hadley LA, Vaughn G, Adams SM, Use of TaqMan real-time reverse transcription–PCR for rapid detection, quantification, and typing of norovirus. J Clin Microbiol. 2006;44:1405–12 . 10.1128/JCM.44.4.1405-1412.2006 16597869 PMC1448641

[9] Nayak MK, Balasubramanian G, Sahoo GC, Bhattacharya R, Vinje J, Kobayashi N, Detection of a novel intergenogroup recombinant norovirus from Kolkata, India. Virology. 2008;377:117–23 . 10.1016/j.virol.2008.04.027 18555887

